# Transtibial Amputation with Removal of the Tibial Intramedullary Nail: Hardware Removal in a Retrograde Manner

**DOI:** 10.1155/2021/6654969

**Published:** 2021-07-19

**Authors:** Youn-Ho Choi, DoJoon Park

**Affiliations:** Department of Orthopaedic Surgery, St. Vincent's Hospital, College of Medicine, The Catholic University of Korea, Seoul, Republic of Korea

## Abstract

Transtibial amputation is the preferred strategy for treating a diabetic foot with an infection and necrosis. However, if a tibial intramedullary nail was previously inserted into the ipsilateral lower extremity, the nail must be removed to perform the transtibial amputation. In this special situation, the removal of the tibial intramedullary nail can cause various complications after transtibial amputation. We present a case and surgical technique report of a 46-year-old male with an uncontrolled diabetic foot with tibial intramedullary nail insertion. With the nail and ankle fixed by distal interlocking screws, a below-knee amputation was performed by removing the nail and the amputated limb together. This surgical method is expected to reduce postoperative complications such as infections and patella instability after the amputation of a diabetic foot.

## 1. Introduction

The below-knee amputation is the most preferred strategy for the surgical treatment for an uncontrolled infected diabetic foot with arteriosclerosis obliterans (ASO) [1–4]. However, in special cases, a surgical plan should be established before performing the below-knee amputation. This study introduces a case of below-knee amputation in an uncontrolled diabetic foot patient who underwent internal fixation with a tibial intramedullary (IM) nail for a tibia shaft fracture. In this case, the IM nail had to be removed to perform the transtibial amputation, so a prepatellar incision was required to remove the IM nail. However, additional incisions during surgery can cause other problems such as surgical site infections and wound disruptions in these patients [5, 6]. In addition, ischemic damage may worsen due to the manipulation applied during IM nail removal. Moreover, since the possibility of patella subluxation or dislocation increases after a below-knee amputation, particular attention may be required in surgery around the knee joint prior to below-knee amputations [7, 8]. Therefore, we described a method of performing a transtibial amputation with IM nail removal in a retrograde manner.

## 2. Case Report

### 2.1. Case Presentation

**Figure 1:**
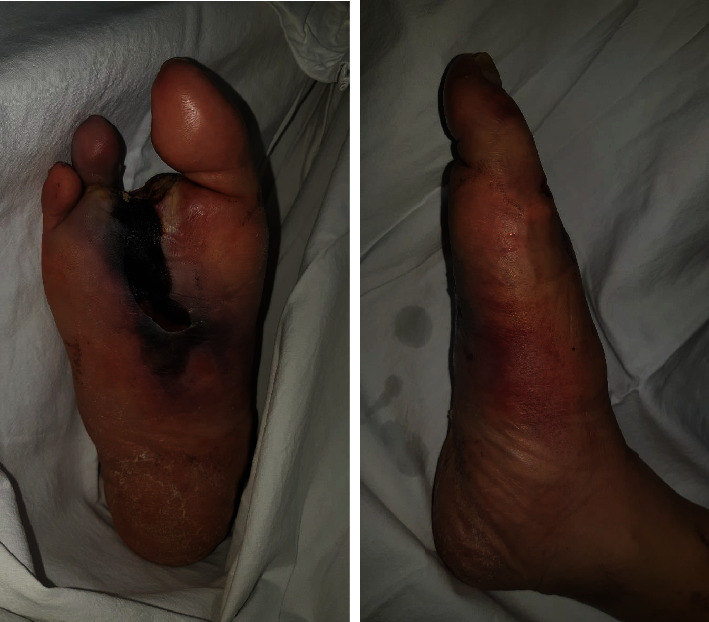
Clinical photographs of the right foot. Active infection and necrosis were limited to the feet.

A 46-year-old man with diabetes mellitus presented to the emergency department at our hospital with symptoms of right foot pain and fever. The patient developed ASO in the right lower extremity as a complication of diabetes. Arterial occlusion started from 10 cm distal to the knee joint and involved the anterior tibial artery, posterior tibial artery, and peroneal artery. The patient had already had right second and third metatarsophalangeal joint disarticulations in the plastic surgery department of our hospital. Nevertheless, infection and necrosis in the right foot progressed rapidly and worsened to a condition requiring additional surgery (Figure 1). However, because a tibial IM nail had been inserted into the ipsilateral limb, the patient was referred to the orthopedic surgery department.

### 2.2. Preoperative Planning

Above-knee and through-knee amputations were also considered surgical options for the patient. However, it was determined that the blood flow to the distal part of the popliteal artery was intact. Also, infection and necrosis had not progressed beyond the foot. The skin color of the right calf seemed healthy on physical examination. Therefore, we decided to remove the tibial IM nail and perform a below-knee amputation. However, performing additional surgery around the knee joint to remove the tibial IM nail was thought to have a high possibility of complications. Thus, surgery was planned to remove the amputated limb and IM nail at the same time by performing an amputation with the nail fixed on the ankle by distal locking screws.

**Figure 2:**
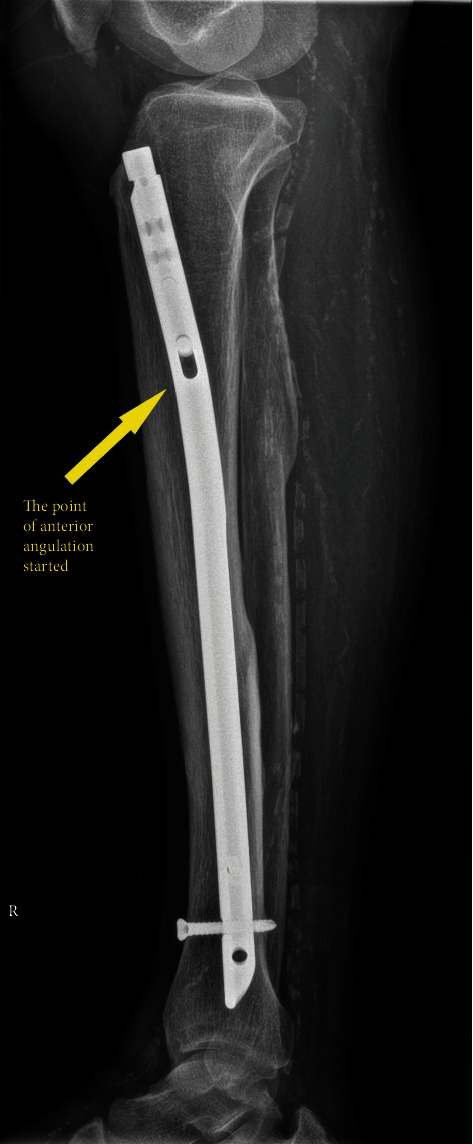
Lateral view of the tibial plain radiograph. The point of the anterior angulation started about 10 cm away from the tibial plateau.

The tibial bone cutting level was determined by referring to the lateral view of the tibial plain radiograph. The tibial IM nail is designed to have an anterior angulation in the proximal part. Therefore, if tibial bone cutting is performed distal to the angulation of the nail, the nail may not be removed from the proximal part of the tibia due to the angulation of the nail. Therefore, we decided to perform tibial bone cutting at the point of the anterior angulation in the nail to leave as much of the tibia as possible (Figure 2). In this patient, the anterior angulation of the tibial nail was about 10 cm away from the tibial plateau on the X-ray, so tibial bone cutting was performed 10 cm away from the tibial plateau.

### 2.3. Surgical Method

**Figure 3:**
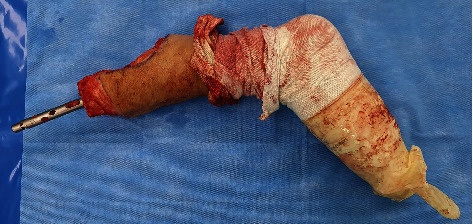
The amputated limb and IM nail were removed from the lower extremities together.

**Figure 4:**
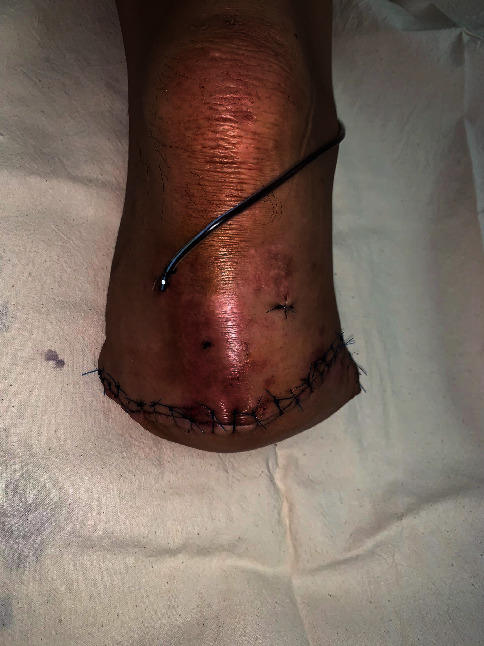
Postoperative photograph of the right lower extremity. No additional incision was applied to remove the IM nail except a mini-incision for the removal of the proximal interlocking screw.

**Figure 5:**
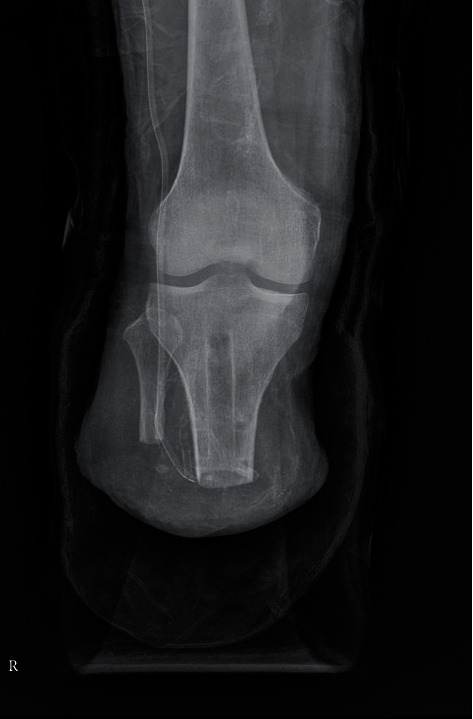
Postoperative plane radiograph. No hardware remained after surgery.

The patient was positioned in a supine position on the operating table. A nonsterile tourniquet was applied to the upper thigh for use in case of major bleeding, and surgery was initiated without inflation of the tourniquet under general anesthesia. Most of the surgery was performed similarly to the typical transtibial amputation procedure, and a long posterior flap was used. The skin incision was designed for a long posterior flap, and an incision was made in the skin and fascia. The anterior and lateral compartment muscles were divided. During dissection, the anterior tibial artery and vein were ligated. The fibula and tibia were exposed, and the proximal interlocking screw of the tibial nail was removed. Among the proximal interlocking screws, the screw located at the distal part was removed from the exposed tibia without an additional incision. The remaining proximal interlocking screw was removed by making a minimal incision of about 7 mm in the previous surgical scar. Next, tibial cutting was performed. Using a ruler, a site about 10 cm away from the tibial plateau was measured and marked on the tibia, and the tibia was cut using an oscillating saw, except the IM nail. A sagittal cutting blade was used for the blade of the oscillating saw. However, the posterior surface of the tibia was cut using a reciprocating blade while protecting the deep posterior compartment with Adson tissue forceps. The fibula was cut 1 cm proximal to the tibia using an oscillating saw and bone cutter. The posterior flap was made by completely separating the posterior musculature from the tibia and fibula. At this point, the amputated limb and lower extremity were connected only by the IM nail inserted into the tibia. Insert an osteotome into the tibial osteotomy gap with a mallet to expand the gap. If the opening of the osteotomy seems insufficient, use 2 or 3 stacked osteotomes or thicker osteotome until the osteotomy is opened to the desired extent. After widening the gap to about 2-3 mm, gentle traction and a twisting force were applied several times on the amputated limb. Then, the amputated limb was separated from the proximal tibia with the IM nail connected (Figure 3). The anterior lip of the tibia was beveled. The tibial, peroneal, and sural nerves were sharply divided and allowed to retract to prevent neuroma formation. The bulky soft tissue of the posterior flap was trimmed to create a properly shaped posterior flap. After irrigation and drainage insertion, the deep fascia and skin were sutured without tension (Figure 4). No hardware remained after surgery (Figure 5).

## 3. Discussion

Below-knee amputations in diabetic patients are still a challenge for surgeons due to the high complication rates [2, 9]. In the case of the patient described in this study, two problems involved with performing tibial nail removal can be summarized. The first problem was wound-related complications. Surgical site infection is the most common complication of surgery in diabetic patients, which adversely affects postoperative outcomes and increases reoperation rates [5]. Also, because most diabetic patients have some degree of microvascular dysfunction, the risk of surgical site disruption is high [6]. Therefore, additional incisions to remove the tibial IM nail increase the rate of surgical wound complications. The second problem is iatrogenic trauma around the knee joint that can occur during IM nail removal. Patients who undergo below-knee amputations are more likely to develop patella subluxation or dislocation. One of the causes is the insufficiency of the medial patellofemoral ligament [8]. Therefore, in these knees, patella subluxation or dislocation can occur with minor trauma [7]. To remove a tibial IM nail, a transpatellar or parapatellar approach is used [10]. However, it is possible to damage the soft tissue around the patella while approaching, and more damage may be caused while manipulating the knee joint to remove the nail. These injuries may increase the possibility of patella dislocation in the future.

In addition to the two causes mentioned above, it might be better to remove the nail in a retrograde manner to minimize the spread of infection from the ankle. If the IM nail is removed by the conventional method, the distal tip of the IM nail and distal locking screws contaminated due to ankle infection may be exposed to the surgical site, thereby increasing the risk of postoperative infection. Due to these negative factors, removing the IM nail prior to performing a below-knee amputation of the limb may lead to undesirable results in infected diabetic foot patients.

The surgical method introduced in this case report did not directly involve the knee joint or the distal part of the lower extremity with the infection source. Therefore, we believe that this surgical method can reduce the risk of postoperative complications when performing below-knee amputations in diabetic foot patients with an IM nail fixation.

## 4. Conclusion

In this case report, the tibial IM nail was not removed at the proximal entry point of the nail but was removed along with the amputated limb. When transtibial amputation is performed on a patient with a tibial IM nail insertion, the tibial IM nail can be removed in a retrograde manner, so it can be considered a surgical option reducing postoperative complications.

## Data Availability

The data that support the findings of this study are available from the corresponding author, Youn-Ho Choi, upon reasonable request.
